# Surveys of post-operative pain management in a teaching hospital in Rwanda — 2013 and 2017

**DOI:** 10.1080/24740527.2019.1673158

**Published:** 2019-10-28

**Authors:** William McKay, Danyela Lee, Adolphe Masu, Shefali Thakore, Eugene Tuyishime, Joseph Niyitegeka, Paulin Ruhato, Theogene Twagirumugabe, Jennifer O’Brien

**Affiliations:** a Department of Anesthesiology, University of Saskatchewan, Saskatoon, Saskatchewan, Canada; b Department of Anesthesiology, Critical Care and Emergency Medicine, University of Rwanda, Kigali, Rwanda

**Keywords:** pain, postoperative, pain management, surveys and questionnaires, income, surgery, perioperative procedures

## Abstract

**Background**: Postoperative pain management (POPM) appeared to be weak in Rwanda.

**Aims**: The aim of this study was to compare POPM measures in a teaching hospital between 2013 and 2017.

**Methods**: A two-phase observational study in 2013 and 2017. was conducted. Participants were recruited prior to major surgery and followed for two postoperative days. A numerical rating scale (0–10) was administered to all participants in both years, and the International Pain Outcomes questionnaire was administered in 2017. Recruitment, consent, and data collection were performed in participants’ preferred language.

**Results**: One hundred adult participants undergoing major general, gynecologic, orthopedic, or urologic surgery were recruited in 2013 and 83 were recruited in 2017. Fourteen percent of participants in 2013 and 46% in 2017 scored their worst pain as severe (>6; *P* < 0.001). This was despite improved preoperative recognition of patients at high risk for severe postoperative pain (those with chronic pain or preoperative pain); 27% and 0% of these patients were not documented in 2013 and 2017, respectively (*P* = 0.006). Other measures of improved planning included “any preoperative discussion of POPM” (*P* < 0.001) and “discussion of POPM options” (*P* = 0.002). Preemptive analgesia use increased (3% of participants in 2013 and 54% in 2017; *P* < 0.001). Incidence of participants having no postoperative analgesic at all decreased from 25% in 2013 to 5% in 2017 (*P* < 0.001).

**Conclusions**: Though severe postoperative pain incidence did not improve from 2013 to 2017, POPM improved by a number of measures. These changes may be attributed to pain research conducted there having raised awareness.

## RÉSUMÉ

**Contexte** : La prise en charge de la douleur postopératoire (POPM) semblait faible au Rwanda.

**Objectifs** : Comparer les mesures de prise en charge de la douleur postopératoire dans un hôpital d’enseignement entre 2013 et 2017.

**Méthodes** : Étude observationnelle en deux phases réalisée en 2013 et 2017; les participants ont été recrutés avant de subir une chirurgie majeure et ont été suivis pendant deux jours après l’opération. Une échelle de notation numérique (0 à 10) a été administrée à tous les participants les deux années, tandis que le questionnaire *International Pain Outcomes* a été administré en 2017. Le recrutement, le consentement et toutes les collectes de données se sont déroulés dans la langue préférée par les participants.

**Résultats** : Cent participants adultes ayant subi une chirurgie générale, gynécologique, orthopédique ou urologique majeure ont été recrutés en 2013; 83 en 2017. Quatorze pour cent des participants ont qualifié leur pire douleur de sévère (> 6) en 2013, et 46 % en 2017 (*p* = 0,001). Cela est survenu malgré l’amélioration de la reconnaissance préopératoire des patients à haut risque de douleur postopératoire sévère (ceux atteints de douleur chronique ou de douleur préopératoire); 27 % de ces patients n’ont pas été documentés en 2013 ; 0 % en 2017, *p* < 0,006. Parmi les autres mesures de planification améliorée, mentionnons « toute discussion préopératoire de la prise en charge de la douleur postopératoire » (*p* < 0,001) et « la discussion des options de prise en charge de la douleur postopératoire » (*p* < 0,002). L’utilisation préventive de l’analgésie a augmenté (3 % des participants en 2013; 54 % en 2017; *p* < 0,001). L’incidence des participants n’ayant reçu aucun analgésique postopératoire est passée de 25 % en 2013 à 5 % en 2017 (*p* < 0,001).

**Conclusions** : Bien que l’incidence de la douleur postopératoire sévère ne se soit pas améliorée de 2013 à 2017, la prise en charge de la douleur postopératoire a été améliorée par un certain nombre de mesures. Ces changements peuvent être attribués au fait que la recherche sur la douleur menée là-bas a permis une plus grande sensibilisation.

## Introduction

“It is estimated today that a lack of adequate pain management affects 80% of the global population, and is a serious problem in over 150 countries.”^1^ We conducted a survey on postoperative pain management (POPM) in the Centre Hospitalier Universitaire de Kigali, the major teaching hospital in Rwanda, in 2013. In 2015, a focus group study involving anesthesia residents was conducted regarding their perspectives, perceptions, and experiences in pain management.^2^ We report an observational study of POPM in patients undergoing major surgery at a Rwandan teaching hospital, consisting of cross-sectional surveys conducted before and after that study, in 2013 and 2017. The rationale for this study arose from the observation that postoperative pain relief in low-income countries (LICs)^*^ ^*^The World Bank classifies countries into four income groups based on gross national income per capita. Currently (2018), the categories in U.S. dollars are as follows:

1. Low-income countries (LICs): $995 or less.
2. Lower–middle-income countries (LMICs): $996 to $3,895
3. Upper–middle-income countries (UMICs): $3,896 to $12,055
4. High-income countries (HICs): >$12,055.

appears poorly developed and managed and of a lower priority than other aspects of health care.^3–5^

In 2012, it appeared that little had changed since the few earlier African studies that all found that POPM was poorly conducted: in 1985 in Nigeria^6^; in 1996 in Nairobi, Kenya^7^; in 1999 in Ibadan, Nigeria^8^, and 2001 a review of results in Ibadan.^5^ These employed a four-step verbal response scale (*none, mild, moderate, severe*). The first study assess pain on this scale once on the day of surgery and again the first day after and found that 38% of patients had moderate pain and 30% had severe pain.^6^ The second used the same scale after major abdominal and thoracic surgery and found that for the first two postoperative days most patients had moderate or severe pain.^7^ The third study found that half of the patients had moderate or severe pain prior to their next dose of analgesia.^8^ Recent studies and studies that track change in pain management are lacking. Good POPM begins in the preoperative period with an assessment of the patient and development of a plan of care tailored to the individual and the surgical procedure involved. It ideally includes organizational policies, perioperative pain management planning, preoperative education, preemptive analgesia, use of appropriate pharmacological and nonpharmacological modalities, and transition to outpatient care.^9^ Preemptive analgesia, an evolving clinical concept, involves the introduction of an analgesic regimen before the onset of noxious stimuli, with the goal of preventing sensitization of the nervous system to subsequent stimuli that could amplify pain.^10^

This project studies progress in the most basic of these, preoperative and postoperative pain assessments, drugs used, and patient perceptions of pain, in Rwanda. The first set of data collected in 2013 is a part of a master of science thesis.^11^

The primary outcome of this project was a comparison of the incidence of severe postoperative pain following major surgery in 2013 and 2017, with a null hypothesis of no change. We also explored changes in POPM practice between the two surveys. Additional questionnaires explored participant attitudes toward pain, expectations, and satisfaction regarding POPM. Secondary outcomes include comparisons of results in 2013 with those in 2017.

## Materials and methods

### Design

Using prospective repeated observational surveys, this study compares POPM methods used in University Central Hospital of Kigali (CHUK) in 2013 to those used in 2017. In order to document changed POPM behavior over time, surveys were undertaken 4 years apart. The dates were chosen for logistical reasons as feasible for scheduling the investigators. The 2013 study, the first prospective survey of POPM in Rwanda, was designed and conducted by local faculty and residents with input from faculty from the University of Saskatchewan. The 2017 study was designed and conducted by faculty and residents from both sites. With approval from the University of Rwanda’s Internal Review Board and the University of Saskatchewan’s Research Ethics Board for both studies, convenience samples of participants were recruited at CHUK. Data were collected in the hospital by trilingual investigators.

CHUK, the main tertiary care referral hospital for Rwanda (population 12 million), has nine operating rooms and 400 beds. Health insurance became mandatory for all individuals in 2008. Details of the health system are outlined in Nyirigira’s 2018 paper.^12^

### Participants

Included were patients who were 18 years of age or older, cognitively able to give informed consent, and scheduled for major surgery. Major surgery was defined as surgery requiring in-hospital care for at least 2 days postoperatively.^13,14^ Recruiting, consent, and all questions were in the participants’ preferred language and always by fluent speakers. Written informed consent was obtained in the language preferred by the participant (Kinyarwanda, French, or English). Rwanda is a small country with a single native language, Kinyarwanda. All native-born Rwandans speak Kinyarwanda; those who have attended primary school also speak French, and those who are university educated also speak English. English was declared an official language in 2018 and is taught in schools. Translations were by the Rwandan authors and approved by the University of Rwanda’s Internal Review Board, whose members are all trilingual, ensuring that translation guidelines were adhered to.^15^

### Measurements

Data were obtained by brief face-to-face interviews that consisted of administering pain scales and questionnaires to the patients, discussing care with the attending nurses, and checking the patients’ charts. Data were entered on paper forms at the patient’s bedside and transferred later to spreadsheets. We recorded patient characteristics (age, sex, type of surgery) and risk factors for severe postoperative pain, namely, chronic pain or severe acute preoperative pain.^16,17^ We asked the participant in the preoperative holding area whether the preoperative evaluation had included a discussion of postoperative pain management. Our clinical experience correlates with the study from Kenya: surgical pain is worst on postoperative days 1 and 2.^7^ Participants were followed on postoperative days 1 and 2 and asked about the presence of preoperative pain (chronic pain or acute injury), severity of pain on a numerical pain scale, whether POPM had been discussed, and whether options had been offered. From the chart and discussion with attending nurses, information was recorded about dose and time of preemptive analgesia, type of anesthesia, whether pain scores were obtained by nursing staff, and all analgesics administered.

Postoperative pain scales were administered in the postoperative care unit (PACU) and on postoperative days 1 and 2. The cross-culturally validated 11-point (0–10) numerical rating scale was selected because it is easily understood by patients and medical staff as well as simple and quick to administer. It is considered valid and reliable and has been used in hundreds of pain studies.^18–20^ The pain scores are conventionally interpreted as follows: 0 = *no pain*; 1–3 = *mild pain*; 4–6 = *moderate pain*; and 7–10 = *severe pain*.^21^ In addition, a brief questionnaire regarding patient attitudes toward pain relief was administered, based on the paper by Scott and Hodson.^22^ Questionnaires were administered and discussed with participants at the bedside. Analgesic doses and times were recorded.

The International Pain Outcomes (IPO) questionnaire was not available in 2013. This resulted in the 2017 cohort being asked about their pain in more detail, which we felt might influence their numerical response scores. For this reason, the IPO questionnaire was administered after pain scores were collected.

Data sheets were stored in locked cabinets and password-protected computers and were used in accordance with the research requirements of the two universities. Participant confidentiality was protected by de-identifying any research-related data through the use of a master list. The master list included patients’ personal heath numbers/hospitalization numbers to anonymize research data. The master list was stored separately from research data in a locked filing cabinet in Dr. Twagirumugabe’s office. The final storage location of the master list and research data was a locked filing cabinet in Dr. Twagirumugabe’s office. No identifiable personal health information left Rwanda; all research data transmitted to Canada for analysis were de-identified.

### Additional measurements

The time the participant entered PACU and the time to first nursing assessment were recorded in 2013. For logistical reasons (in 2013 the data were collected by A.M., who was available to go to the PACU; much of the data in 2017 were collected by OR nurses, who could not leave their other duties to do this task), this was not done in 2017. In 2017, the IPO (Appendix 2) was administered on postoperative day 2. This instrument, validated for use in multiple languages, covers five aspects of outcome measurements for acute pain: pain severity, interference with function, affective experience, side effects, and perceptions of care. It is intended as a tool for overall assessment of the quality of acute pain management. It was not available for the first study, because it was published after our Research Ethics Board submission was made.^23^

### Statistical analysis

A convenience sample of 100 patients was chosen as likely obtainable in the time available. The sociodemographic characteristics of participants were analyzed using descriptive statistics. We report means and standard deviations for normally distributed variables, medians, and quartiles for nonnormally distributed variables and frequencies for categorical variables. We report the continuous ^†^ ^†^Although pain scale data are ordinal, there is a long tradition of reporting pain as continuous. and categorical outcomes of the administered pain scale. Where appropriate, we compare the findings of 2013 with those of 2017 with *t* tests for normally distributed continuous data, Mann-Whitney *U* tests for data that were not normally distributed, and chi-square test for proportions and for Likert scale distributions. Sidak correction was made for multiple comparisons; with nine comparisons, the alpha level was adjusted to 0.006. Findings other than the primary outcome should be considered hypothesis generating. Through the informal questionnaires, we drew themes of value for future study and for educational purposes.

## Results

For the primary outcome, the incidence of severe postoperative pain (worst pain score >6) following major surgery, the null hypothesis of no change from 2013 to 2017 is refuted. The expected change was a decrease in incidence of severe pain, but we were surprised to find an increase in the percentage of patients with severe pain from 4% (95% confidence interval, −0.5 to 13) to 45% (95% confidence interval, 35 to 57; *P* < 0.001).

Patient characteristics are reported in Table 1, with caregiver behaviours and analgesics in Table 2. Sex distribution was not different (*P* = 0.08), but the distribution of types of surgery changed, with more gynecology and fewer orthopedic cases (*P* = 0.04).

**Table 1.** Demographics.

	2013 (*n* = 100)	2017 (*n* = 83)	*P* value
Age (years), mean (SD)	40 (16)	36 (11)	NS
Male	52 (52%)	35 (38%)	NS
Female	48 (48%)	55 (62%)	
Surgery type			
General surgery	38	35	NS
Obstetrics and gynecology	19	32	
Orthopedics	40	22	
Urology	3	2	
Type of anesthetic			
General	64 (64%)	47 (57%)	NS
Intrathecal	36 (36%)	36 (43%)	
Postoperative pain added risk identified^a^			
Yes	12 (12%)	31 (37%)	
No	61 (61%)	52 (63%)	NS
Not assessed	27 (27%)	0 (0%)	<0.001

^a^ Participants with chronic or severe acute pain preoperatively.

There was an increase in intraoperative morphine use (33% to 51%, *P* = 0.01) as well as an increase in dose. There was no difference between years in doses or frequency of intraoperative fentanyl use (103 ± 14 μg). All patients receiving general anesthesia got one or both. Ketamine was not used intraoperatively in 2013, but 43% of patients received 100 mg in 2017. Participants were all Rwandans; foreigners tend to go to a nearby private hospital. One hundred participants were recruited from June to August 2013. All participants were followed on the ward. Due to time constraints posed by the investigators’ schedules, only 91 participants were recruited in November and December 2017. Of these, only 83 could be followed postoperatively on the ward because one died, five were discharged on the day of surgery (thus disqualifying them as having undergone major surgery), one was admitted to the intensive care ward too heavily sedated to communicate, and one patient’s surgery was postponed. Day 1 pain scores are reported in Table 2, caregiver behaviors and analgesics in Table 3, questionnaire results in Table 4, and IPO questionnaire scores in Table 5.

**Table 2.** Scores for worst postoperative pain.

	Year	0	1–3	4–6	7–10	*P* value^a^
Day 1 pain score distribution	2013	7	22	18	3	<0.001
	2017	0	5	40	38	
		Number	Median	25th Percentile	75th Percentile	
Day 1 score statistics	2013	50	3	2	5	<0.001
	2017	83	6	5	8	

^a^ Chi-square test.

**Table 3.** Caregiver behaviors and analgesics used.

Preoperative	2013 (*n* = 100)	2017 (*n* = 83)	*P* value
Discussed POPM	0/100 (0%)	13/83 (16%)	<0.001
Discussed POPM options	0/100 (0%)	9/83 (11%)	0.002
Preemptive analgesia	3/100 (3%)	45/83 (54%)	<0.001
Intraoperative analgesics (*n*; median [interquartile range])			
GA morphine (mg)	33; 4 [4 to 5]	42; 5 [5 to 5]	0.01
GA fentanyl (μg)	39; 100 [100 to 100]	43; 100 [100 to 100]	NS
GA ketamine (mg)	0; 0 [0 to 0]	35; 100 [60 to 150]	<0.001
Intrathecal morphine (μg)	25; 200 [200 to 200]	6; 100 [100 to 100]	<0.001
Intrathecal fentanyl (μg)	3; 100 [100 to 100]	27; 20 [20 to 20]	<0.001
Postoperative			
Time until seen in PACU (min) mean (SD) [range]	61 (56) [10 to >120]		
Pain score taken in PACU	9 (9%)		
Pain score taken on day 1	44 (44%)	83 (100%)	<0.001
Pain score taken on day 2	40 (40%)	83 (100%)	<0.001
POPM medication^a^			
Morphine	4 (4%)	29 (35%)	<0.001
Tramadol	36 (36%)	36 (43%)	NS
Diclofenac	22 (22%)	14 (17%)	NS
Acetaminophen	4 (4%)	20 (24%)	<0.001
Ibuprofen	2 (2%)		NS
Fentanyl	0	1 (1%)	NS
None	16 (16%)	4 (5%)	0.03

^a^ Postoperatively on the ward.

POPM = postoperative pain management; GA = general anesthetic; PACU = postoperative care unit.

**Table 4.** Participant expectations—Questionnaire results.

		None	Mild	Moderate	Severe	Excruciating	*P* value^a^
Participant expectation of pain	2013	3	10	34	25	0	<0.001
	2017	16	31	17	14	5	
Likert scale		*Strongly agree*	*Agree*	*Neutral*	*Disagree*	*Strongly disagree*	
I need pain to heal	2013	0	9	3	31	0	<0.001
	2017	3	13	7	39	20	
I need to tell the nurse about my pain	2013	0	59	12	1	0	<0.001
	2017	12	51	7	9	4	
The nurses responded to my pain	2013	0	59	12	1	0	<0.001
	2017	1	3	11	12	56	
Despite my pain, I was satisfied with my care	2013	0	71	1	0	0	<0.001
	2017	57	19	3	3	1	

^a^ Chi-square test.

**Table 5.** 2017 IPO summary data.

		Numerical response scale score (0–10) or %			
	Brief description	Mean	SD	Min	Max
P1	Worst pain since surgery	6.3	1.8	3	10
P2	Least pain since surgery	2.9	1.5	1	7
P3	Percentage of time in severe pain	44.2	21.9	10	100
	Pain interfered with or prevented:				
P4a	Moving in bed	5.0	2.0	1	9
P4b	Deep breathing and coughing	2.7	3.0	0	10
P4c	Sleep	2.7	2.5	0	9
P4d-ii	Out-of-bed activities	3.5	2.0	0	9
	Pain made you feel:				
P5a	Anxious	2.6	2.8	0	9
P5b	Helpless	1.0	2.0	0	7
	Postoperative side effects:				
P6a	Nausea	2.3	2.9	0	9
P6b	Drowsiness	2.2	2.5	0	9
P6c	Itching	0.9	2.0	0	10
P6d	Dizziness	2.3	2.9	0	10
P7 (%)	Percentage of pain relief since surgery	54.2	21.3	10	100
P10	I was involved in pain decisions	0.1	0.4	0	3
P11	Satisfied with pain treatment	7.4	2.1	0	10
P13a	If you had chronic pain, how severe was it?	6.5	2.2	3	10

Despite apparently improved treatment, pain was rated by participants as significantly higher in 2017 than in 2013 (median [25th to 75th percentile] = 3 [2–5] in 2013; median = 6 [5–8] in 2017; *P* < 0.001).

### 2013

Additional findings from 2013 are that preemptive analgesics were very rarely used for major surgery (3 of 100 participants); the time taken by PACU nurses to do their first assessment of newly admitted participants was unacceptably long by the standards of high-income countries (HICs), with a mean time of 1 hour (longest time >2 hours, and formal pain scores in PACU were rarely taken by PACU nurses (9 of 100). On the wards, POPM administration was unacceptably low by HIC standards but was dramatically improved over the 4 years. In 2013 fewer than half of participants had formal pain scores on the wards on postoperative day 1 or 2. Only 29% had postoperative opioid analgesic after major surgery, low by HIC practice, and 25% had no postoperative analgesic at all.

### 2017

By 2017, all of the measurements except severe pain had significantly improved. Although participants in 2017 clearly expected significant postoperative pain, they no longer believed that pain is necessary, natural, and hence beneficial, as suggested in an earlier paper (Table 5).^4^

## Discussion

### Main findings

Reporting of severe postoperative pain increased despite apparent improvement in pain management behaviors.

Of the criteria for good POPM management enumerated in the widely accepted 2016 guidelines,^9^ preoperative assessment of the patient for added pain risk improved from 12% to 37%, and preoperative discussion of pain as a first step in development of a plan of care tailored to the individual and the surgical procedure involved improved from 0% to 16%. Little or no preoperative pain planning and assessment led to inadequate POPM in 2013, but both aspects of pain management were improving by 2017. The introduction of ketamine during anesthesia was a major change in practice.

An unexpected finding was that in 2013 the pain scores were lower than those in 2017 (*P* < .001), with only three participants complaining of severe pain in 2013. That some patients had no postoperative analgesia at all (25% in 2013, 5% in 2017; *P* = 0.03) would be unacceptable by HIC practice; the incidence of this was much improved but still occurred.

### Previous studies

Although statistical comparison is not possible, the 2017 scores on day 1 appear to be more comparable to those of an HIC and to those of the standard care group in an international multicenter trial that included participants from India, a low–middle-income country (Table 6).^24^

**Table 6.** Comparison of pain scores with other studies of major surgery.

Study	*n*	Median	Lower quartile	Upper quartile	Range
Kigali 2013	50	3	2	5	0 to 7
Kigali 2017	83	6	5	8	3 to 10
Saskatoon 2007^39^	37	4.9	3.6	7.2	1 to 10
Sweden 2003^40^	442	5.2	3	7	0 to 10
International 2017^24^	222	4.5	2.7	7	

### Suggested explanations

The improvement in POPM management by caregivers pre- and postoperatively is likely attributable to increased caregiver awareness of POPM as an issue. This may be due in part to increased global awareness and in large part from local research activities in CHUK.^4^ A qualitative study of anesthesia resident knowledge and practice was conducted in CHUK in 2012 and began to increase awareness in the anesthesia department.^2^ Between our surveys, two further POPM studies were conducted by our group in the operating rooms and surgical wards at CHUK.^25,26^ Resident awareness of POPM as an integral part of patient care was heightened over the years from 2010 to 2018. This is borne out by anesthesia faculty members, Dr. Gaston Nyirigira and coauthor Dr. Eugene Tuyishime, who trained during that period and who initiated the first international postoperative pain control educational meeting (Zero Pain Rwanda), held in Rwanda in January 2019.

The worsening of pain scores cannot be explained by differences in age, gender, ethnicity, or surgery mix (Table 3). Despite more intrathecal morphine and fentanyl use in 2013, the introduction of ketamine and increased morphine use in 2017 make it unlikely to be due to altered anesthesia practice. Although we do not have data to support or refute this, a possibility lies in the trend in HICs of treating more surgery as suitable for discharge home on the day of the operation.^27^ This could mean more that participants with less severe pain were admitted for 2 days in 2013 who would have been sent home early in 2017. We suspect that the new emphasis on POPM by nursing changed the ward culture and allowed patients to complain who would never have done so in 2013. This borne out by the fact that all seven patients in 2013 whose worst postoperative pain was scored zero were men, suggesting a cultural need to appear tough. Future studies with the IPO will help to clarify this.

### New knowledge

Documenting and monitoring a clinical problem are necessary but not sufficient steps toward improving it.^28^ The few surveys of LIC postoperative pain management as well as attitudes and expectations showed little improvement over almost 30 years. The findings of our 2013 survey are not dramatically different from the earliest of these from 1985 but have improved considerably since.^6–8,29–32^ Our studies suggest that though occasional surveys of POPM have had little effect, combining them with a POPM research program raises awareness and may be more effective in changing behaviors.

### Weaknesses

A weakness of the study is that it was done in one hospital in one LIC and thus should not be considered generalizable. Nonetheless, it may signal some even worse POPM in other LIC hospitals, because CHUK is the major teaching and referral hospital in Rwanda. Another weakness is that the two surveys are not identical. The IPO was not available at the time of application for ethics approval for the first survey. We plan to include it in future surveys.

A potential weakness may result from using length of stay in defining major surgery as surgery requiring admission for 2 days. Hospital stay in Rwanda may not reflect only the type of surgery. Patients may stay longer due to inability to pay the small hospital bills they have incurred. However, though it is true that length of stay does not depend entirely on clinical discharge criteria, the fact that patients are admitted to a surgical ward means that the surgery is sufficiently major to require inpatient nursing care, at least for a day or two. Patients having minor procedures such as excision of a small skin lesion are not admitted to hospital.

The pain scale and questionnaires are not specifically validated for Rwanda, although cross-cultural validation was performed.^20^ However, 71% of Rwandan adults are literate, and we found that they understood the study and the questions.^33^

Part of the reason for missing data comes from the sometimes fraught experience of conducting clinical research in LICs. The 2013 application to the University of Rwanda and University of Saskatchewan research ethics boards included these words: “Quantitatively, we will be measuring pain scores serially postoperatively as well as other measures of pain.” An anesthesia resident from the University of Saskatchewan (coauthor S.T.) was funded by the University of Saskatchewan College of Medicine to go to Rwanda and collaborate with her Rwandan counterpart (coauthor A.M.), but following an Ebola outbreak in neighboring Uganda, the College of Medicine would not permit her to go for reasons of safety. In the ensuing flurry of communications, A.M. thought that we were to measure *the incidence of pain score recording by caregivers*, until we met online to discuss the progress of the study at the 6-week mark, by which time he had recruited 76 participants. As a result, we had pain scores for 50 of the 100 participants in 2013.

As stated, a convenience sample of 100 patients was chosen as likely obtainable in the time available. It has been suggested that we should have used the scores from the first study to calculate sample size for the second. Post hoc calculation with a conservative expected difference of 1 on the numerical rating scale yields *n* = 38 with power = 0.8 and alpha = 0.05, so the sample size was adequate.^34^

### Strengths

To help avoid missing tasks done but not charted and finding those charted but not done, our study was conducted primarily at the bedside with the patient, with examination of what was charted for secondary findings.

The IPO questionnaire^26^ has been validated mainly for European and Israeli populations. No current studies exist for the validation of the IPO questionnaire in any African population. In addition, the questionnaire developed by Scott and Hodson^27^ is not a validated screening tool and was used primarily in a North American population and thus may not appropriately reflect the attitudes or cultural differences of the patients being treated at the Kigali Health Institute. To address these limitations, we addressed face validity by sending both the IPO and the Scott and Hodson questionnaire to nonclinical staff within the Kigali Health Institute to determine how easily the questions were understood and completed and to note any difficulties or ambiguities with items in the questionnaires. We amended question wording to ensure that patients would be able to understand and complete the questionnaires appropriately.

### Past and future studies

Pain appeared anecdotally to be inadequately treated in the teaching hospitals of Rwanda as noted in meetings (W.M., A.M., P.B., T.T.) in 2010. Since those discussions, our group has implemented a research program to improve POPM in CHUK consisting of the first survey reported here (2013), followed by a dose-finding study of low-dose subcutaneous ketamine (2015),^25^ a randomized clinical trial of low-dose subcutaneous ketamine (2016),^26^ the 2017 survey, and meetings to discuss implementation of the Essential Pain Management™ program at CHUK (2018). A survey of pediatric POPM is approved for 2019. A follow-up adult survey is planned for 2020, which will follow implementation of a comprehensive POPM system (Essential Pain Management).^35^

### Conclusions

The widespread lack of effective pain management is an ethical issue involving the principle of beneficence as reported in the mission statements of professional organizations.^4^ Suggested reasons for a lower standard of POPM in LICs include patient expectations, caregiver expectations, and limited resources. Patients expect surgical pain, fear opioids, and have a poor understanding of the benefits of pain relief.^3–5^ Lack of education among health care providers regarding pain, its physiology, and its management may contribute to suboptimal pain management.^2^ Resources for pain management are also limited: the pharmacy at CHUK is not as reliably supplied with medications as those in HICs, and overworked ward nurses may be engaged with more urgent issues as they care for large numbers of patients. Despite the complexity of the problem of poor POPM, improvement is likely with increased awareness.

We are uncertain why pain scores were lower in 2013 than in 2017. The effect of recruiting more gynecology and fewer orthopedic cases is uncertain. Increased awareness among staff of POPM deficiencies may have been unconsciously communicated to patients.

Severe postoperative pain, though improving, is still too common in CHUK. Good POPM improves surgical outcomes.^36^ Mechanisms include improved mobility, with its many benefits, including prevention of deep vein thrombosis and pneumonia; improved psychological outcomes including anxiety and depressive mood; improved social outcomes such as quality of life and independence and strain on family relations; and improved organizational outcomes including length of stay, mortality, and cost. Cardiovascular, respiratory, and gastrointestinal systems are adversely affected by poor pain management. Unrelieved pain after surgery increases catecholamine levels, which then increase heart rate and systemic vascular resistance, which may place the patient at risk of myocardial infarction.^37^ Unrelieved acute postoperative pain may lead to chronic pain conditions.^38^

Our findings show that though many measures of POPM are improving, we need to continue to improve POPM in Rwanda. More resources need to be provided for POPM, including teaching of surgical, anesthetic, and nursing staff; strategies to improve continuity of drug supply to hospitals; and patient education. We trust that these surveys will help CHUK and other LIC hospitals to prioritize the planning and implementation of the components of good POPM enumerated in the Introduction.
